# Protein Glycosylation in *Helicobacter pylori*: Beyond the Flagellins?

**DOI:** 10.1371/journal.pone.0025722

**Published:** 2011-09-30

**Authors:** Patrick S. Hopf, Rachel S. Ford, Najwa Zebian, Alexandra Merkx-Jacques, Somalinga Vijayakumar, Dinath Ratnayake, Jacqueline Hayworth, Carole Creuzenet

**Affiliations:** Infectious Diseases Research Group, Department of Microbiology and Immunology, The University of Western Ontario, London, Ontario, Canada; Institut de Pharmacologie et de Biologie Structurale, France

## Abstract

Glycosylation of flagellins by pseudaminic acid is required for virulence in *Helicobacter pylori*. We demonstrate that, in *H. pylori,* glycosylation extends to proteins other than flagellins and to sugars other than pseudaminic acid. Several candidate glycoproteins distinct from the flagellins were detected via ProQ-emerald staining and DIG- or biotin- hydrazide labeling of the soluble and outer membrane fractions of wild-type *H. pylori*, suggesting that protein glycosylation is not limited to the flagellins. DIG-hydrazide labeling of proteins from pseudaminic acid biosynthesis pathway mutants showed that the glycosylation of some glycoproteins is not dependent on the pseudaminic acid glycosylation pathway, indicating the existence of a novel glycosylation pathway. Fractions enriched in glycoprotein candidates by ion exchange chromatography were used to extract the sugars by acid hydrolysis. High performance anion exchange chromatography with pulsed amperometric detection revealed characteristic monosaccharide peaks in these extracts. The monosaccharides were then identified by LC-ESI-MS/MS. The spectra are consistent with sugars such as 5,7-diacetamido-3,5,7,9-tetradeoxy-L-glycero-L-*manno*-nonulosonic acid (Pse5Ac7Ac) previously described on flagellins, 5-acetamidino-7-acetamido-3,5,7,9-tetradeoxy-L-glycero-L-*manno*-nonulosonic acid (Pse5Am7Ac), bacillosamine derivatives and a potential legionaminic acid derivative (Leg5AmNMe7Ac) which were not previously identified in *H. pylori*. These data open the way to the study of the mechanism and role of protein glycosylation on protein function and virulence in *H. pylori.*

## Introduction

*Helicobacter pylori* chronically infects 50% of the world's population and causes gastritis, gastric ulcers and cancers [1], [2]. There is a ∼6-fold increased risk of gastric cancer after *H. pylori* infection, and gastric cancer is the second most common cause of death from cancer [3]. Therefore, *H. pylori* causes a huge burden on the economy and health care system. The efficacy of current treatments is threatened by the emergence of antibiotic resistance [4], [5]. Hence, novel treatments are urgently needed. Abundant research has identified a large array of virulence factors in *H. pylori*, including lipopolysaccharide, adhesins, toxins, urease, the Cag pathogenicity island and flagella (Reviewed in [6]). The flagellins (FlaA, FlaB) that make up the flagellar filament are O-glycosylated, and their glycosylation is essential for flagella production and virulence [7], [8], [9], [10]. Likewise, glycosylation of the flagellins occurs and is also necessary for flagella formation in the closely related *Campylobacter jejuni* [11], [12], [13]. Protein glycosylation has now been described for many other prokaryotic proteins. While the precise role of glycosylation on the function of bacterial proteins is not well understood, glycosylation appears to contribute to the virulence of a number of pathogens [14], [15], [16], [17], [18], [19], [20] and potentially to tolerance in human symbionts [21].

Bacterial protein glycosylation is very diverse in terms of the size, composition and structure of the oligosaccharides present, and the sugars can be derived from O-antigen synthesis pathways or stem from dedicated pathways (Reviewed in [22]). The cellular location and mechanism of glycosylation also vary, encompassing transfer of complex glycans onto their target protein in the periplasm, or stepwise addition of single sugars in the cytoplasm.

To date, *C. jejuni* is the only known bacterium with both N- and O- protein glycosylation pathways. The O-glycosylation pathway targets the flagellins that are modified by pseudaminic acid (PA) derivatives and is conserved in *H. pylori* [13], [23], [24]. In contrast, the N-glycosylation pathway appears unique and was proposed to glycosylate ∼38 *C. jejuni* proteins by a diacetamidobacillosamine (DAB)-containing heptasaccharide [25], [26]. Both pathways have been characterized at the biochemical level [23], [24], [27], [28], [29], [30], [31], [32], [33]. Of relevance to this study, the PA biosynthesis pathway is initiated by the UDP-GlcNAc C6 dehydratase FlaA1 (HP0840) and the aminotransferase HP0366.

While abundant literature is available on protein glycosylation in *C. jejuni*, little is known about glycosylation of proteins other than flagellins in *H. pylori*. We observed that inactivation of the PA pathway by disruption of the *flaA1* gene affects virulence factor production beyond the lack of flagellum [10]. In this manuscript, we show that this is also the case upon disruption of *hp0366*. This suggests that, in contrast to the current dogma, PA-dependent protein glycosylation is not limited to the flagellins in *H. pylori.* Our data suggest that the PA pathway also targets proteins other than the flagellins. Furthermore, our data indicate that a novel PA-independent glycosylation pathway exists in *H. pylori* and glycosylates numerous proteins. Mass spectrometry analyses performed on sugars extracted from candidate glycoproteins provided hits consistent with novel carbohydrates not previously identified in *H. pylori*. Combined with the identification of the novel glycoprotein candidates, this work will eventually also allow us to determine the role of glycosylation on protein function, an area that has remained elusive for bacterial proteins.

## Results

### Disruption of the PA biosynthesis pathway affects the production of multiple virulence factors

**Figure 1:**
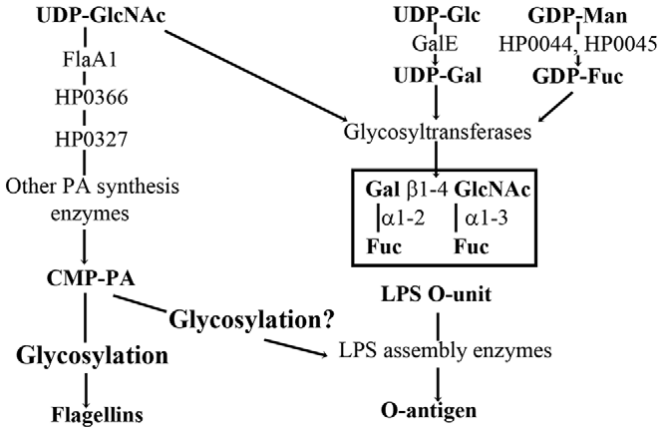
Schematic representation of key steps of the PA and LPS biosynthesis pathways. The main enzymes responsible for the synthesis of sugar nucleotides are indicated, including the dehydratase FlaA1 and the aminotransferase HP0366 relevant to this work. The links between both pathways are highlighted, namely a shared precursor UDP-GlcNAc, and the potential glycosylation of LPS assembly enzymes by PA.

**Figure 2:**
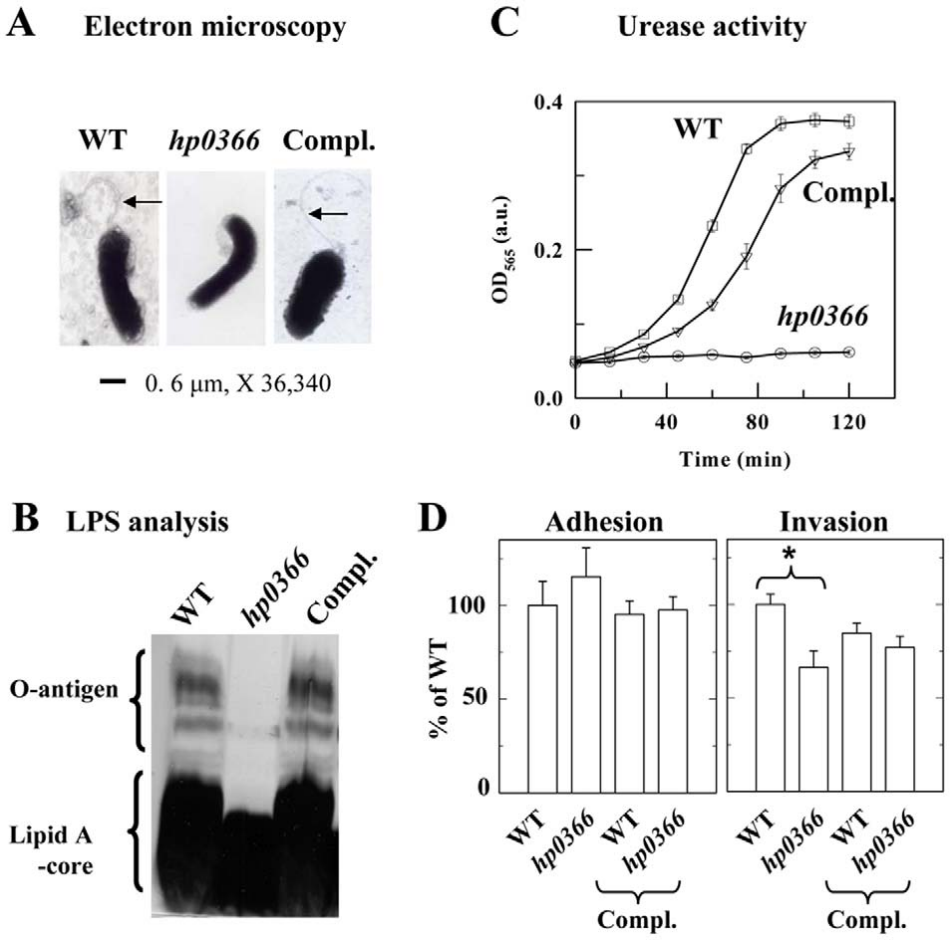
Impact of disruption of *hp0366* on virulence factor production and interactions with gastric cells. Panel A: Analysis of flagellum production by electron microscopy. Arrows point at flagella. **Panel B:** Analysis of LPS production. LPS was extracted from the WT, mutant and complemented strain (Compl.) and analyzed by SDS-PAGE and silver staining. **Panel C:** Analysis of the urease activity of WT, mutant and complemented (Compl.) *H. pylori*. Urease activity was measured using phenol red as an indicator [92]. Experiments were done with 3 different concentrations of soluble protein extracts and the same trend was observed. The data shown were obtained with the highest amount of protein tested and are the average of three replicas. **Panel D:** Adherence and invasion of *H. pylori* WT, mutant and complemented strains to gastric AGS cells. The data are expressed in % of adherence or invasion of WT *H. pylori*. The data are the average of 3 independent experiments. Compl. indicates that the wild-type *hp0366* gene was introduced in the strain of interest on a shuttle plasmid. The adherence of WT amounted to 8–10% of the inoculum. No statistical differences (t-test) were observed between WT and mutant strain for adherence. Invasion was measured after elimination of non-internalized bacteria by gentamycin treatment. Invasion represented ∼1.5% of the inoculum for the WT. Statistical differences were observed between the WT and mutant (shown by asterisk, t-test, p<0.001).

Mutants with a disrupted PA biosynthesis pathway were constructed to determine the impact of the PA pathway on the production of non-flagellar virulence factors. The *flaA1* mutant, in which the first step in PA biosynthesis is disrupted (Fig 1), was described previously [10]. A *hp0366* mutant, in which the second step of the PA pathway is disrupted (Fig 1), was constructed for this study in a similar manner, via insertion of a kanamycin resistance cassette in *hp0366*. This mutant grew at rates comparable with wild-type (WT) (Data not shown). Like the *flaA1* mutant, the *hp0366* mutant was non-motile (Data not shown) and did not produce flagella (Fig 2A) although it still produced flagellins (Data not shown). Inactivation of *flaA1* or *hp0366* also affected LPS synthesis. Specifically, the *flaA1* mutant produced altered O-antigen [10] and the *hp0366* mutant lacked O-antigen (Fig 2B). This is despite the fact that the O-antigen sugars of the lipopolysaccharide (LPS) do not comprise PA and are synthesized by enzymes distinct from FlaA1 and HP0366 (Fig 1) [34], [35], [36], [37], [38], [39]. Also, both mutants had reduced urease activity ((Fig 2C and [10]) as demonstrated by direct comparison of the OD_565nm_ values obtained for the wild-type and mutant extracts used at the same concentrations. All phenotypes were gene-specific and could be complemented by introduction of the corresponding gene *in trans* (Fig 2 and [10]).

Furthermore, *H. pylori* is known to adhere to and invade gastric cells [40], [41], [42], [43]. Adhesion leads to alterations of cellular signaling that are important for pathogenicity [44], [45], [46]. In *C. jejuni*, protein glycosylation affects interactions of the bacterium with intestinal cells [47], [48]. Therefore the impact of inactivation of *hp0366* on adhesion and invasion of host cells was tested. Under our experimental conditions, ∼8% and 1.5% of the WT inoculum adhered to and invaded gastric cells, respectively. These data are on par with the literature and reflect the fact that invasion is very inefficient [49], [50]. Inactivation of *hp0366* resulted in reduced invasion of gastric AGS cells, while no effect was observed on adherence (Fig 2D). Under the conditions used for adhesion and invasion assays, urease activity and flagella-mediated activity do not play any role since a neutral pH is maintained throughout the experiments and the bacteria are forced to make contact with the epithelial cells via centrifugation. Furthermore, the lack of effect of *hp0366* disruption on adhesion indicates that the flagella do not serve as adhesins. Therefore, the disruption of the PA pathway, and not the lack of flagellum assembly, appears to be directly responsible for the differences seen in bacterial invasion in the *hp0366* mutant. Although the reason is not clear, the introduction of the complementation plasmid in the WT strain led to decreased invasion. Therefore, complementation for this phenotype was assessed by comparing the levels of invasion of the WT and mutant strains, each harboring the complementation vector. No significant differences could be observed between the two complemented strains, whereas statistically significant differences were observed between the original strains.

Altogether, these data indicate that, in *H. pylori*, the PA pathway is essential for the production of multiple virulence factors (urease, LPS) beyond the flagellum, and is also important for interactions with host cells. The fact that the *flaA1* and *hp0366* mutants, that both lack assembled flagella but have disruptions at different steps of the PA pathway, present different phenotypes in terms of LPS synthesis suggests that the effects observed on virulence factor production are related to inactivation of the PA pathway rather than to the lack of flagellum assembly.

### Pleiotropic or transcriptional effects do not explain the multiple effects of the PA pathway on production of virulence factors

Beyond the previously reported lack of membrane-associated UreA and UreB in the *flaA1* mutant [10], no significant differences were observed in the inner and outer membrane proteins of the *flaA1* and *hp0366* mutants compared with the wild-type (WT) (Data not shown). This indicates that the differences of production of surface-associated virulence factors are not due to pleiotropic effects that would have resulted in the instability of the bacterial cell wall.

Also, the coordinated impact of PA synthesis disruption on several apparently independent virulence factors may involve transcriptional regulation. In *H. pylori*, joint transcriptional regulation of urease and flagellum production occurs via the flagellar *flbA* gene [51], [52], and *hp0366* belongs to the *flbA* regulon [53]. Therefore, we measured the levels of expression of genes involved in virulence factor production in the *flaA1* and *hp0366* mutants by quantitative real time PCR (qRT-PCR). The genes tested comprised genes for urease and its accessory proteins, LPS biosynthetic enzymes (synthases, transferases, O-antigen ligase) and the flagellar regulator FlbA.

**Figure 3:**
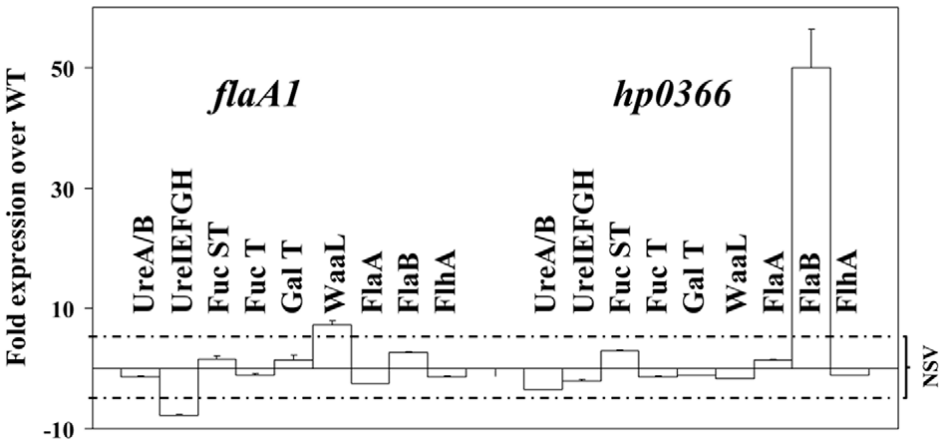
qRT-PCR analysis of the impact of the *flaA1* or *hp0366* inactivation on transcription of genes involved in urease, LPS and flagellum production. All data were normalized to the level of expression of the housekeeping gene ***hp1045*** **, the acetyl-CoA synthase.** Differences are considered significant for >5 fold difference compared with WT. The interval for non significant variation (NSV) is delimited by dotted lines. Error bars are for duplicate experiments done using the same sample of RNA. The same experiment was also repeated on RNA diluted 1/10 (v/v) and the same trend was observed (not shown). UreA/B: operon encoding both UreA and UreB. UreIEFGH: operon encoding 5 urease accessory proteins. FucST: fucose synthase. FucT: fucose transferase. GalT: galactose transferase. WaaL: O-antigen ligase. FlaA and FlaB: flagellins A and B. FlhA: flagella basal body and transcriptional regulator, also known as FlbA.

Concerning urease production, the only significant variation was a slight down-regulation of the urease accessory gene cluster observed in the *flaA1* mutant only (Fig 3), while both mutants show reduced urease enzymatic activity. Therefore, transcriptional regulation does not explain fully the decrease in urease activity of the two mutants. Concerning LPS biosynthetic genes, no significant decrease was observed in the transcription of any of the genes tested in the *hp0366* mutant, and a slight up-regulation of the O-antigen ligase gene *waaL* was observed in the *flaA1* mutant only. Therefore, transcriptional regulation does not explain the lack of O-antigen of the *hp0366* mutant or the reduced amount of O-antigen of the *flaA1* mutant. Finally, for the flagellar genes, there were no transcriptional differences in the *flaA1* mutant, and there was a ∼50 fold up-regulation of *flaB* in the *hp0366* mutant, which, if anything, should contribute to enhanced flagellum production. This does not correlate with the observation that both mutants were aflagellate. Note that the differential transcriptional regulation of *flaA* and *flaB* observed in the *hp0366* mutant is consistent with the fact that flagellin genes are under the control of different alternative sigma factors: sigma 28 for FlaA and sigma 54 for FlaB [53].

Overall, these data suggest that transcriptional regulation is not causing the observed interconnection of flagella, LPS and urease production in the PA mutants. In light of the fact that the observed phenotypes of the *flaA1* and *hp0366* mutants are gene specific and do not result from pleiotropic nor transcriptional regulatory effects, we proposed that the PA pathway may glycosylate proteins involved in the production of virulence factors other than the flagellins, and that their glycosylation is necessary for their function. These include, for example, enzymes involved in LPS assembly (Fig 1). Therefore, the experiments described below were aimed at demonstrating the existence of such proteins, and characterizing their carbohydrate content.

### ProQ-emerald staining suggests that *H. pylori* produces numerous glycoproteins

**Figure 4:**
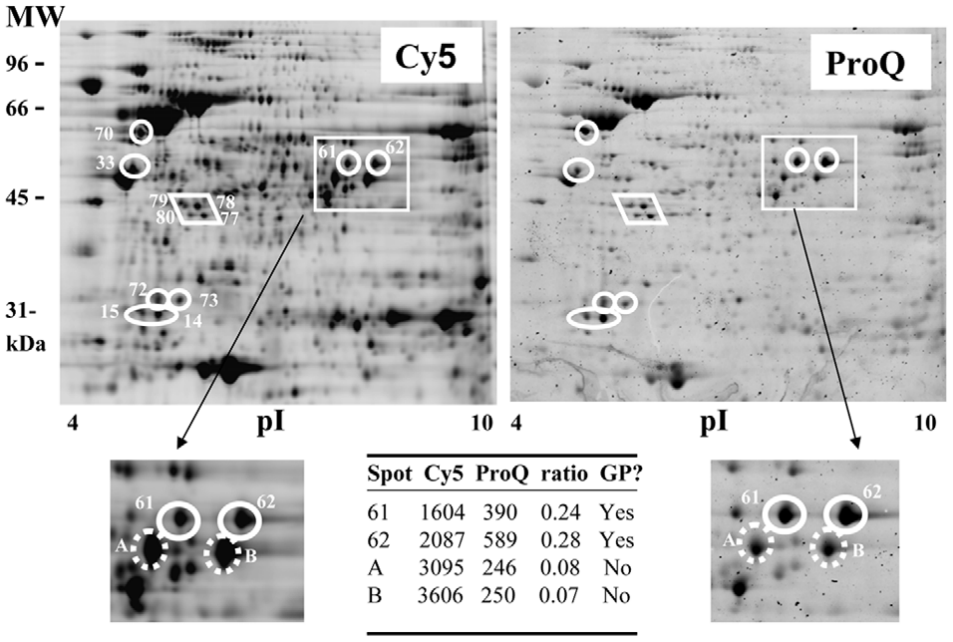
2D gel electrophoresis analysis of *H. pylori* glycoproteins using Cy5 labeling of total proteins and ProQ-emerald labeling of glycoproteins. The proteins from WT *H. pylori* were stained with Cy5 and were resolved by 2D gel electrophoresis. Glycoproteins were stained with ProQ-emerald. Abundant proteins gave a high background reactivity by ProQ-emerald labeling (ex: UreB). To eliminate false positive proteins, the ratio of the ProQ-emerald and Cy5 signals was calculated and only proteins that showed a high ratio were considered GP candidates. The ratios calculated are indicated for a few spots shown as an example in the zoomed figures. Spots A and B are provided as examples of non glycosylated proteins. Signals for Cy5 and ProQ-emerald are in arbitrary units. Contributions from the gel background have already been subtracted. Note that the analysis was limited to a subset of 100 proteins that were present in sufficient amounts to allow their identification by MS ultimately. The 12 spots highlighted on the figure (in circles and diamond) are the ones with the highest ratios in this subset and represent GPs. Additional GPs may be present.

To investigate whether *H. pylori* produces additional glycoproteins (GPs), the total proteins were labeled with the fluorescent Cy5 dye, resolved by 2D SDS-PAGE, and the GPs were detected by ProQ-emerald staining after oxidation of the sugars (Fig 4). While the *H. pylori* genome only encodes ∼1600 ORFs, 2,300 protein spots were detected by Cy5 labeling. This suggests that numerous proteins exist in multiple isoforms due to post-translational modifications. Out of these, 756 spots were detected using the ProQ-emerald reagent. The fact that some abundant proteins did not react with the ProQ-emerald stain whereas some low abundance proteins did react indicates that the ProQ-emerald reagent is specific for glycoproteins. However several highly abundant proteins reacted non-specifically with ProQ-emerald, likely due to the stringent conditions used for the oxidation of the sugars. To eliminate false positive proteins, the ratio of intensities of the ProQ-emerald and Cy5 signals was calculated for a subset of 100 proteins that were present in high enough amount (based on Cy5 signal) to allow downstream identification by MS. Spots that had very low ratios (0 – 0.09) correspond to very weakly ProQ-emerald labeled proteins that probably stained in a non specific manner and were not considered for further analysis. These represented the vast majority of the spots analyzed (∼80%). When the distribution of the ProQ/Cy5 ratios of all other spots was plotted, a bimodal distribution was observed, with a group of spots exhibiting ProQ/Cy5 ratios <0.16 and another group with ratios between 0.17 and 0.33. The spots showing intermediate ratios (0.10–0.16, first group) could potentially correspond to proteins glycosylated at very low levels. The higher ratios (0.17 and 0.33) observed in the last group of spots suggest a higher level of glycosylation of these candidate glycoproteins. Therefore, only proteins with a high ProQ/Cy5 ratio (>0.17) were considered GP candidates worthy of further investigation (See Table insert in Fig 3 for example). A t-Test indicated a significant difference (p<0.001) between these GP candidates (mean ProQ/Cy5 ratio 0.2335) and the non GP proteins (mean ProQ/Cy5 ratio 0.1125) using this threshold value of 0.17.

This method revealed the existence of 12 candidate GPs (Fig 4). Their identities as obtained by MALDI-MS are listed in Table 2. Most of the hits ran at a higher molecular weight (∼0.2 to ∼7.2 kDa) than predicted based on their amino acid sequence, consistent with post translational modification. Note that the same hit was often obtained for two independent and well resolved spots, which is consistent with the heterogeneity of protein glycosylation that can give rise to multiple protein isoforms. Also, two strong hits were obtained for each of spots 14 and 15, including a common hit for the putative FliT flagellar chaperone. Overall, this analysis led to the identification of 9 GP candidates within the 12 ProQ-emerald-reactive spots.

The ProQ-emerald-based discovery of 9 potential GPs suggests that glycosylation is not limited to the flagellins. This warrants further investigation of these GP candidates and of their sugar content, since some of the detected proteins may contain a single sugar while others may contain multiple reacting sugars at multiple protein sites. While concomitant identification of GPs and their glycans is possible by MS directly from 2D gel protein spots [54], [55], [56], interpretation of the data is very complex for bacterial GPs where the nature of the sugars and their organization is not known. Furthermore, the GP candidates highlighted by ProQ-emerald staining are not very abundant proteins. Therefore, we employed a cellular fractionation strategy to facilitate the recovery of larger amounts of putative GPs and allow the characterization of their sugars.

### Cellular fractionation shows that the soluble and outer membrane fractions both contain GP candidates

**Figure 5:**
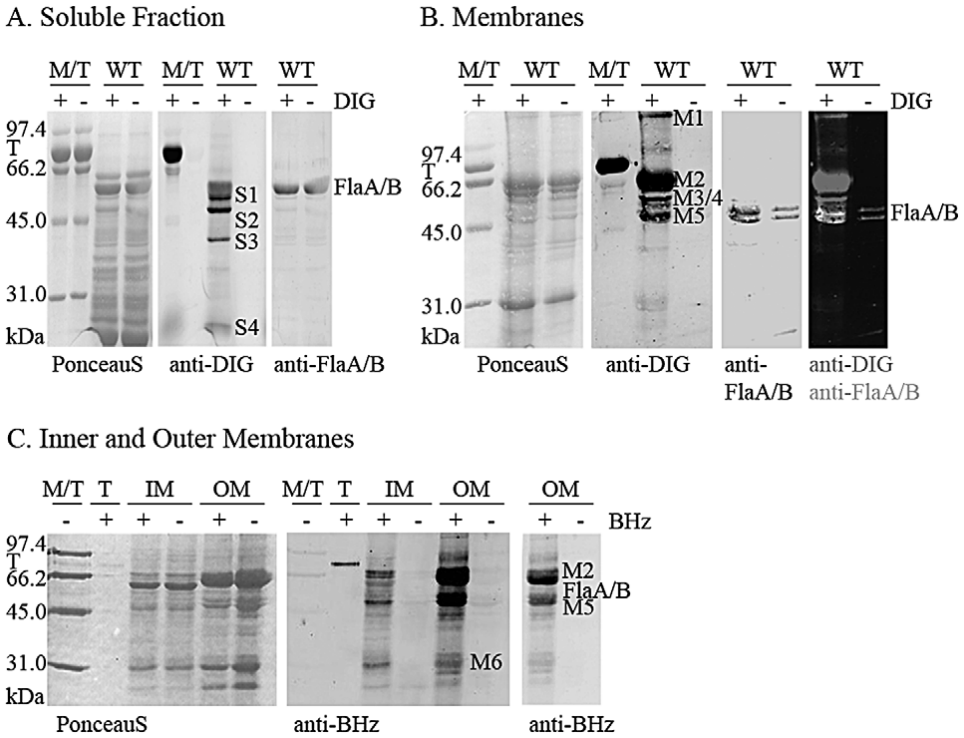
Analysis of the GPs from the soluble and membrane fractions of the WT *H. pylori* strain by SDS-PAGE gel and DIG labeling. The cells were lysed by mechanical disruption, separated into the soluble (Panel A) and membrane (Panel B) fractions by ultracentrifugation. The membrane fraction was also separated in its inner and outer membrane components (Panel C, IM and OM, respectively). The proteins were labeled with DIG- (Panels A and B) or biotin- (panel C) hydrazide (+) or not (−), and separated on a SDS-PAGE gel. The GPs were detected by anti-DIG Western blotting (Panels A and B, red in overlay) or streptavidin Western blotting (Panel C). An anti-flagellin Western blot (green in overlay) was also performed to show that most GPs detected are not flagellins. M/T: molecular weight marker in which the glycoprotein transferrin was added to serve as a positive control for the DIG/Biotin blots. T: transferrin only. BHz: biotin hydrazide. The most DIG/biotin-reactive GPs are labeled on each panel. The anti-biotin blot of the OM is provided at two levels of exposure to allow visualization of the low reactivity band M6 and the doublet constituting the high reactivity band M2.

Total proteins were separated into their soluble and membrane complements by ultracentrifugation, and the presence of putative GPs was assessed in each fraction by digoxigenin-3-O-succinly-ε-aminocaproic acid hydrazide (DIG) labeling after periodate oxidation [57]. The oxidation step generates carbonyls from sugars that contain vicinal alcohol groups. The DIG-hydrazide reacts with the carbonyls that were formed during the oxidation step, or with free carbonyls that may be present in some sugars prior to oxidation. The reaction between the carbonyls and the hydrazide results in covalent labeling of the sugars (and therefore of the glycoproteins) by DIG. The DIG label was detected by anti-DIG Western blotting. An anti-flagellin Western blot was also performed to determine whether non-flagellin GPs were present. This analysis showed the presence of DIG-reactive candidate GPs other than flagellins, each in the soluble and total membrane fractions (Fig 5A bands S1–S4 and Fig 5B bands M1 to M5). Similarly to the ProQ-emerald staining, the intensity of the DIG reaction may vary with the degree of protein glycosylation and with the type of sugar present on the candidate GP.

Fractionation of the membrane fraction in the inner and outer membranes (IM and OM, respectively) was performed by differential solubilization of the inner membrane with lauryl sarcosine [58]. This method has been successfully employed for *H. pylori* [59], [60], [61]. Efficient separation was indicated by the different protein patterns of both fractions as assessed by Ponceau red staining (Fig 5C), the detection of LPS Lewis Y O-antigens in the OM fraction only (Data not shown), and the detection of activity of the IM marker lactate dehydrogenase [62] mostly in the IM fraction (Data not shown).

The candidate GPs were labeled with biotin-hydrazide instead of DIG-hydrazide (commercial supply discontinued during the course of our studies). The principle of labeling is identical to that of DIG-hydrazide so that the data are directly comparable. Biotin-hydrazide labeling showed that most of the detectable membrane GP candidates were observed in the OM (Fig 5C). The OM GPs included the flagellins, which was consistent with the presence of a membranous sheath around the flagella [63]. The other abundant and highly reactive GP candidates present in the OM had molecular weights (MW) corresponding to M2 and M5, where M2 appeared as a doublet. M5 appears different from the flagellins as it migrates in between the two flagellin bands (Fig 4C). In addition, a candidate GP that was barely detectable in total membranes appeared enriched in the separate membrane fractions (see band M6). To ascertain that the biotin reactivity observed in the OM samples arose from sugars comprised in glycoproteins and not from other sugars such as the lipopolysaccharides, the OM samples were treated with proteinase K prior to biotin labeling. In this case, no signal was observed upon anti-biotin Western blotting (Data not shown).

In the IM fraction, reactivity was also observed at MW corresponding to bands M2 and M5. These may correspond to additional candidate GPs, or may represent low amounts of contamination of the IM fraction by OM proteins despite the multiple rounds of solubilization and ultracentrifugation performed to separate both membrane fractions, and overall efficient separation achieved.

### Existence of several protein glycosylation pathways in *H. pylori*

The first indication that the PA biosynthesis pathway may not be the only protein glycosylation pathway in *H. pylori* was obtained by comparative analysis of the 2D ProQ-stained GP patterns of the WT and PA pathway mutant, *hp0366*. The global GP profiles appeared identical (Data not shown), and the ProQ/Cy5 ratios of our candidate GPs were not significantly altered by inactivation of *hp0366* (Table 2, unpaired t-Test p value >0.05 when comparing the ProQ/Cy5 ratios of GP candidates in wild-type and *hp0366* mutant). The only exceptions were the two spots that comprised GP candidate #9, whose ProQ/Cy5 ratios decreased 1.5 to 2 folds, respectively, indicating that the glycosylation of GP #9 may be HP0366-dependent.

**Figure 6:**
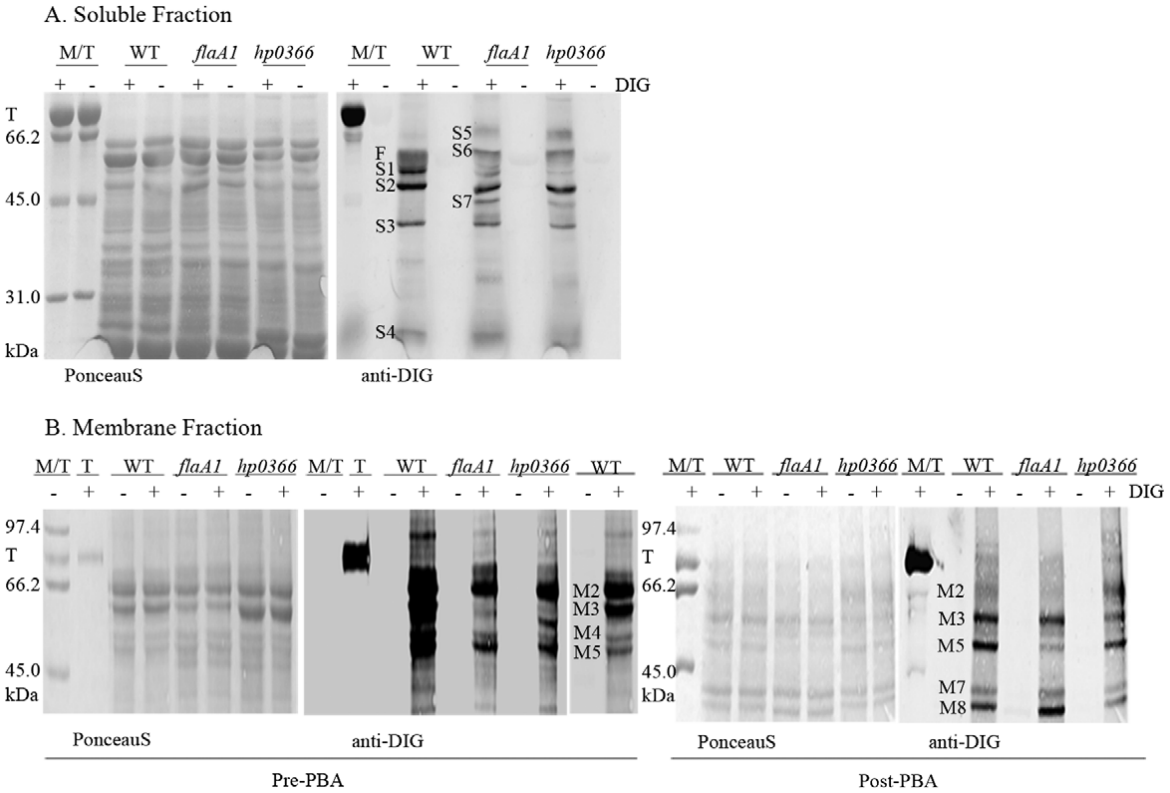
Comparative analysis of the GPs of the soluble and membrane fractions between the WT and *flaA1* and *hp0366* mutants. **Panel A**: soluble proteins. **Panel B**: membrane proteins. The soluble and membrane fractions were prepared as described in **Figure 4**. The membrane fractions were run through a phenyl boronic acid (PBA) column (Post-PBA) or not (Pre-PBA) before DIG labeling and SDS-PAGE analysis. Because of the higher DIG-reactivity of the WT membrane fraction compared with the mutants despite equal protein loadings, the WT is shown at two different levels of exposure to allow identification of the bands. For all panels: T: transferrin. F: flagellins. +: DIG labeling. −: no DIG labeling.

Because the stringent oxidation conditions used for ProQ-emerald staining of 2D gels generate background signal which may mask low abundance GP candidates, comparative analysis of GP profiles of WT and *flaA1* and *hp0366* mutants was also performed using DIG-hydrazide labeling after cellular fractionation. Analysis of soluble fractions of WT and *flaA1* and *hp0366* mutants via DIG labeling demonstrated the existence of candidate GPs whose glycosylation is not affected by disruption of the PA pathway (Fig 6A bands S2–S4). This suggests that a PA-independent glycosylation pathway exists in *H. pylori*. In addition, this analysis revealed that the glycosylation of at least one abundant and soluble candidate GP is affected by disruption of the PA pathway (Fig 6A, band S1). This PA-dependent band does not correspond to any of the flagellins based on anti-flagellin Western blotting. This suggests that the PA glycosylation pathway targets at least one non-flagellar soluble GP in *H. pylori*. Finally, three soluble GP candidates not previously detected in the wild-type strain were detected in the PA mutants (Fig 6A, bands S5, S6, S7). The appearance of these bands suggests co-regulation or cross-talk between the PA-dependent and PA-independent pathways highlighted, so that disruption of the PA pathway increases production of specific GPs via the PA-independent pathway.

**Table 2:** Identification of candidate glycoproteins by ProQ-emerald staining.

GP #	Spot #(MW^a^)	ProQ/Cy5WT	ProQ/Cy5*hp0366*	ORF	Function	%^b^	MW^b^	N-glyc sequon^d^
1	14	0.25	0.19	HP1588	Conserved unknown	51	28.4	None
2	(28.5)			HP0879	Flagellar chaperone FliT	37	28.3	2, G
3	15	0.25	0.19	HP1043	Transcriptional regulator	64	25.5	1, G
2	(28.5)			HP0879	Flagellar chaperone FliT	53	28.3	2, G
4	33	nd	nd	HP1132	F0F1 ATP synthase subunit	73	51.5	1, G
	(52.1)				β			
5	61	0.24	0.25	HP0026	Citrate synthase GltA	44	48.3	1, G
	(53.6)							
5	62	0.28	0.28	HP0026	Citrate synthase GltA	49	48.3	1, G
	(53.6)							
6	70	0.21	0.18	HP1134	F0F1 ATP synthase subunit	32	55.1	1, G
	(62.3)				α			
7	72	0.19	0.17	HP0900	Hydrogenase HypB	51	27.3	1, G
	(30.6)							
7	73	0.20	0.20	HP0900	Hydrogenase HypB	40	27.3	1, G
	(30.6)							
8	77	0.33	0.23	HP1037	X-Pro dipeptidase	32	40.8	None
	(42.6)							
8	80	0.19	0.22	HP1037	X-Pro dipeptidase	44	40.8	None
	(42.6)							
9	78	0.20	0.15	HP1555	Elongation factor EF-Ts	54	39.7	None
	(44.3)							
9	79	0.24	0.13	HP1555	Elongation factor EF-Ts	50	39.7	None
	(44.3)							

Total proteins were labeled with Cy5, resolved by 2D gel electrophoresis and glycoproteins were labeled by ProQ-emerald staining. The ratios of ProQ-emerald to Cy5 staining of the wild-type and *hp0366* mutant strains and the protein identities are indicated for the 12 spots identified as GP candidates so far. Because the same protein hits were obtained for different spots, this results in the identification of 9 GP candidates. The spot numbers refer to Figure 3.

a average molecular weight calculated from 2 independent ProQ-emerald-stained gels.

b % coverage of the protein sequence for MS identification.

c molecular weight as per amino acid sequence, in the absence of glycosylation.

d N-glyc sequon: each ORF was examined for the presence of a N-glycosylation sequon of the general NxS/T type or extended bacterial type D/ExNxS/T where x is any amino acid except proline. The number of sequons is indicated, as well as their type: G, for general.

nd: ratio not determined due to smear from abundant neighbouring spot upon Cy5 detection.

Indications for the existence of several glycosylation pathways were also obtained from the analysis of the membrane fractions. First, disruption of the PA pathway results in a significantly reduced DIG-hydrazide reactivity of the membrane GP candidates at equal protein loadings, indicating that the PA pathway targets several membrane proteins. Second, several GP candidates were still present in the mutants, suggesting that their glycosylation is not PA dependent. Third, an altered candidate GP profile was obtained upon purification of the wild-type membrane GPs by phenyl boronate affinity chromatography (PBA), which is specific for sugars containing vicinal diols (Fig 5B). Namely, some DIG-reactive material was not recovered after passage through the PBA matrix. The differences observed in the pre- and post- PBA fractions suggest that the GP candidates carry different sugars that have different affinity for the PBA matrix. This implies the existence of several glycosylation pathways in *H. pylori.* Specifically, bands M2 and M4 were not (or only poorly) recovered after PBA chromatography while bands M3 and M5 were consistently recovered. Interestingly, the PBA chromatography allowed enrichment of the samples in two candidate GPs (M7 and M8) that were not apparent by analysis of total membranes or even by analysis of IM or OM fractions. Globally, our analysis of the membranes reveals the existence of 8 GP candidates in addition to the 2 flagellins, and indicates that their sugar complement may be different based on differential reactivity with the PBA matrix.

Lastly, bands M3, M7 and M8 were present in the PBA elutions of the membrane fractions of the PA mutants, indicating that they likely arise from a PA-independent pathway (Fig 6B). In contrast, less signal was observed for band M5 in the *flaA1* mutant but not in the *hp0366* mutant (Fig 6B). This suggests the existence of an alternate glycosylation pathway that branches off downstream of FlaA1 but upstream of HP0366.

In summary, these data highlight that *H. pylori* may have 3 glycosylation pathways: the PA pathway, a PA-independent pathway, and a third pathway that branches off the PA pathway downstream of FlaA1. This third pathway may simply reflect the existence of a redundant aminotransferase activity for the PA pathway in *H. pylori*. Moreover, these data suggest that the PA pathway also targets non flagellar proteins.

### Identification of DIG- or Biotin-reactive GPs

**Figure 7:**
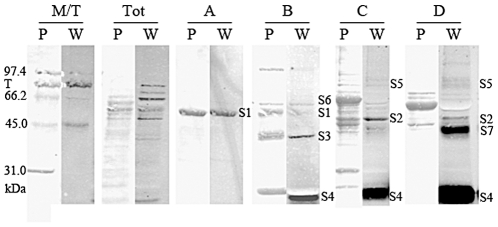
SDS-PAGE analysis of anion exchange chromatography fractions that contain the most DIG-reactive GPs. The fractions were screened by Dot-blot and SDS-PAGE by DIG labelling and anti-DIG Western blotting, and the fractions A, B, C and D shown here contain the most DIG-reactive GPs. Tot: total soluble proteins before ion exchange chromatography. M: Molecular weight marker. T: transferrin. P: Ponceau red staining of total proteins. W: anti-DIG Western blot. S1 to S4 correspond to the GPs detected on Figure 4A, while S5 to S7 correspond to GPs that are only detectable in WT after enrichment of the samples by ion exchange chromatography. However, these GPs were detectable in the total soluble fraction of the *flaA1* and *hp0366* mutants.

To identify the soluble DIG-reactive GP candidates, the proteins were first separated by anion exchange chromatography, and the unbound proteins were then separated on a cation exchange chromatography column. The fractions were screened for their DIG- or Biotin- reactivity by dot-blot and Western blot. This yielded 4 different anion exchange chromatography fractions (A to D) that contained 1 to 4 DIG-reactive GP candidates each (Fig 7). Fractions C and D contained a GP candidate that had the expected molecular weight (MW) of one of the PA-independent band S2 (∼50 kDa, Fig 6A). Fractions A and B contained a candidate GP that had the expected MW of the PA-dependent band S1 (∼52 kDa, Fig 6A). This suggests that these bands comprised several non resolved candidate GPs, or may contain one GP glycosylated at varying degrees. This is possible since, as seen for flagellins, glycosylation does not always cause a significant MW variation. Also, a few DIG-reactive bands (S4 and S7) did not stain with Ponceau S red, suggesting that these proteins are present in trace amounts but may be heavily glycosylated.

Identification of the GP candidates by MS analysis of excised and trypsinolyzed DIG-reactive bands from the ion exchange fractions mentioned above was attempted for 4 of the 7 soluble GP candidate bands. This led to 2–3 protein hits per band, reflecting the presence of multiple proteins in each band. For example, band S2 shown in fraction C on Fig 7 provided hits for the general F0F1 ATPase (β subunit) and for glutamine synthase with similar coverage. Therefore, the definitive identification of these candidates will require direct analysis of their glycopeptides, which is beyond the scope of this study.

**Figure 8:**
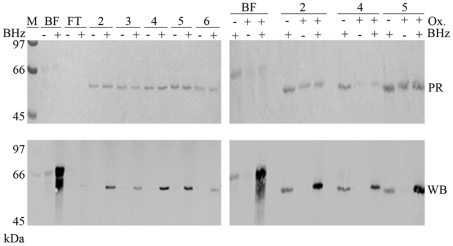
SDS-PAGE analysis of cation exchange chromatography fractions that contain a highly biotin-hydrazide reactive GP. **Left panel**: The fractions obtained by analysing the flow through of the anion exchange chromatography column by cation exchange chromatography were labelled with Biotin-hydrazide and analysed by SDS-PAGE with Ponceau staining, followed by Streptavidin Western blotting. This revealed the presence of a single highly reactive glycoprotein. This protein was identified as catalase by MS analysis of bands shown in fractions 2 to 6. **Right panel**: Fractions showing the highest Biotin-hydrazide reactivity on Panel A (fractions 2, 4 and 5) were tested for non-specific reactivity in the presence or absence of periodate oxidation. This showed that periodate oxidation is necessary to obtain full biotin-hydrazine reactivity, therefore demonstrating that reactivity is due to the presence of sugars on the protein. M: Molecular weight marker. BF: bovine fetuine, used as glycoprotein control. FT: flow through of the cation exchange column. Numbers 2 to 6: cation exchange fractions. PR: Ponceau red staining. WB: streptavidin Western blot.

The cation exchange fractions all contained only 2 bands (at ∼62 and 65 kDa) when analyzed by 1D-SDS-PAGE and Coomassie staining (Data not shown). The most abundant band (at ∼62 kDa) was observed over a wide range of eluting NaCl concentrations and was highly reactive with Biotin hydrazide (Fig 8, left panel). Based on its migration, this band potentially corresponds to band S6 observed previously in the mutants, which may have been masked by the high reactivity of the nearby flagellin band in total soluble protein fractions of the wild-type strain (Fig 6A). Cation exchange chromatography fractions collected at different NaCl concentrations along the elution gradient were run on 1D SDS-PAGE gels, and the biotin-hydrazide-reactive band was excised and identified by MS as catalase in all fractions analyzed (HP0875, MW 58,7 kDa, 60–65% coverage). No other protein hit was obtained. The very strong biotin-hydrazide reactivity observed in all fractions indicate that catalase may be glycosylated and that its glycosylation may be heterogeneous. The difference between the calculated MW and the observed MW (∼62kDa) has been observed previously with catalase over-expressed in *E. coli* [64] and is therefore not due to glycosylation but to anomalous migration on SDS-PAGE gels. Because catalase is involved in resistance to oxidative stress, it could be the target of non specific oxidation that would give rise to non specific glyco-staining. This non specific staining would occur in the absence of the periodate oxidation step that is used to oxidize the sugars. To ascertain that this was not the case, we compared the levels of biotin-hydrazide labeling obtained in the absence or presence of a periodate oxidation step. We observed that the signals obtained after periodate oxidation of the sugars were significantly greater in all fractions compared with the background reactivity present in the absence of periodate oxidation (Fig 8, right panel). Therefore, we can conclude that catalase is a glycoprotein candidate that exhibits heterogeneous glycosylation.

### Demonstrating the presence of sugars on the DIG-reactive soluble GP candidates by HPAE-PAD

**Figure 9:**
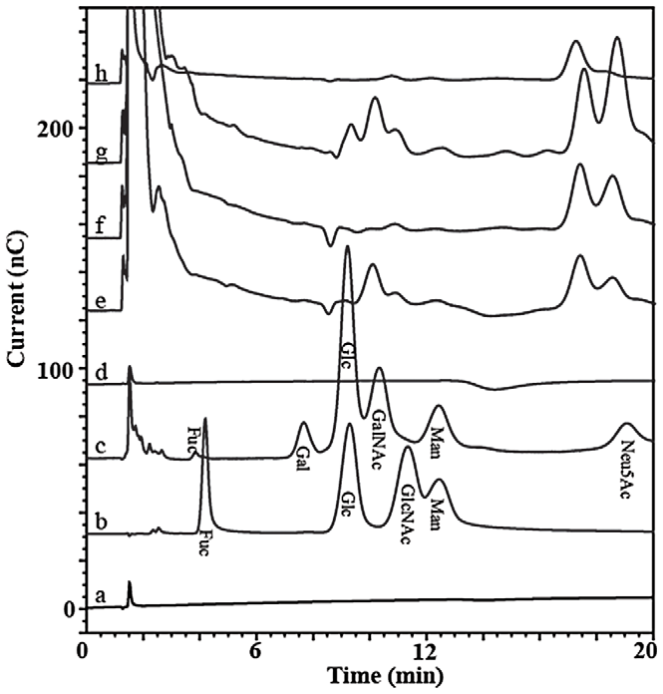
HPAE-PAD analysis of sugars released from GPs by acid treatment. Controls included the baseline (trace a), sugar standards (trace b), and acid extracts from a known glycoprotein (bovine fetuine, trace c) or from a non-DIG reactive ion exchange protein fraction (trace d). Acid extracts from DIG-reactive ion exchange protein fractions presented several potential sugar peaks (traces e to h for fractions D, C, B and A, respectively).

Similarly to the ProQ-emerald staining, highly abundant proteins may react non-specifically with DIG hydrazide due to the stringent conditions used for oxidation of the sugars. Likewise, highly oxidized proteins may also react non -specifically. Therefore, demonstration that the protein fractions analyzed by DIG Western blotting comprise GPs requires direct demonstration that the glycans are covalently attached to the proteins. Such analyses are complex when both the GP candidates and the sugars are unknown. To facilitate these future analyses, the sugars present in these fractions were extracted and identified. The glycans were released from each anion exchange chromatography protein fraction mentioned above by acid hydrolysis [65], [66] and the proteins were eliminated by ultrafiltration. The released sugars were separated on a High Performance CarboPac PA1 anion exchange column [67] and detected by pulse amperometric detection (HPAE-PAD) [68]. The HPAE-PAD profiles clearly confirmed the presence of sugars (Fig 9), with 2 to 5 peaks observed in each of the DIG-reactive fractions analyzed. No peaks were observed upon analysis of protein fractions that did not react with DIG, therefore confirming that the peaks arise from the sugars carried on the proteins present in the ion exchange chromatography fractions. These data represent the first demonstration that *H. pylori* proteins other than flagellins carry sugars. Moreover, the sugar profiles obtained were different from one fraction to the other, indicating that the proteins analyzed have different glycosylation patterns. This also supports the hypothesis that *H. pylori* possesses several glycosylation pathways. The sugars could not be identified from these data for lack of known standards. Therefore, the sugar samples were subjected to mass spectrometry (MS) analysis for identification as described below.

### Identification of the sugars carried by the DIG-reactive soluble GP candidates by MS

The HPAE-PAD method described above does not allow the identification of novel sugars for which standards are not available. Therefore, the sugars were analyzed by LC-ESI-MS/MS. Data analysis was performed using the GlycoWorkBench software [69], which allows manual input of desired sugars in the search list. The expected molecular weights of PA and of other unusual bacterial sugars, such as legionaminic acid and bacillosamine derivatives [26], [70] were added to the search list. Validation of the data analysis system was obtained using *C. jejuni* flagellins, which were purified in a soluble form (as opposed to *H. pylori* flagellins that are membrane-associated) and are glycosylated by PA derivatives similarly to the *H. pylori* flagellins. Hits for the expected PA derivatives were obtained with this control sample (Data not shown).

**Figure 10:**
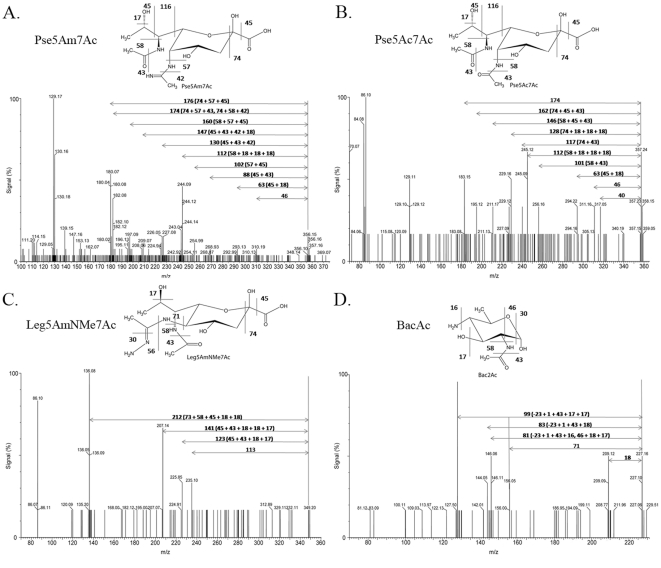
Mass spectrometry analysis of the sugars extracted from GP candidates by acid hydrolysis. The sugars were analyzed by LC-MS/MS. In each panel, the molecular structure of the expected sugar is shown above the MS/MS spectrum, with the expected fragmentation pattern and associated mass loss. The MS/MS spectra are annotated with the total mass loss intervals and with mass loss combinations that lead to the size of the observed peaks. All spectra are shown as sodium adducts. Panel A: Pse5Am7Ac. Panel B: Pse5Ac7Ac. Panel C: Leg5AmNMe7Ac. Panel D: Bac2Ac.

As for the *H. pylori* GP candidate samples, hits were obtained for two pseudaminic acid derivatives (Pse5Ac7Ac and Pse5Am7Ac) in fraction A, and the tandem MS analysis patterns were consistent with this assignment (Fig 10 A and B). Fraction A also had hit whose MS/MS pattern was consistent with a legionaminic acid derivative Leg5AmNMe7Ac (Fig 10C). As well, hits were obtained for two bacillosamine derivatives (Bac2Ac and Bac2Ac4Ac) in fraction D and the monoacetylated form was confirmed by MS/MS analysis (Fig 10D). The concomitant presence of hexose was also demonstrated by MS/MS in this fraction (Fig S1). Note that Pse and Bac derivatives are known to arise from distinct biosynthetic pathways in *C. jejuni* [27], [28], [30], [32], therefore further supporting our conclusion that *H. pylori* has several glycosylation pathways.

Finally, hits with MS/MS patterns consistent with 3-deoxy-D-*manno*-octulosonic acid (Kdo) were also obtained for fractions C and D (Fig S1). Kdo is typically exclusively found in the LPS that is associated with the OM. These fractions did not contain any fucose, a signature component of *H. pylori* LPS. The soluble fraction where Kdo was detected was obtained after two rounds of ultracentrifugation of the cellular lysate to eliminate membrane components. The supernatant was further subjected to ion exchange chromatography and ammonium sulfate precipitation prior to release of the sugars from GP-containing fractions for MS analyses. Therefore, the presence of contaminating sugars arising from the LPS of the OM is highly unlikely. This was nevertheless assessed by SDS-PAGE and silver staining after subjecting the fractions to proteinase K treatment under conditions typically used to prepare LPS [71]. No traces of LPS could be detected by this method. Therefore, the Kdo likely originates from glycoproteins.

Overall, sugars could be extracted from our enriched soluble glycoprotein fractions that were DIG-reactive but not from non DIG-reactive fractions, and the MS/MS patterns of these sugars were consistent with novel sugars never shown to be present in *H. pylori* before. The preparation of the fractions involved extensive removal of membrane components by repeated ultra-centrifugation steps, as well as selective precipitation of proteins by ammonium sulfate, followed by ion exchange chromatography. Therefore, the extracted sugars were originally protein-associated. These data clearly demonstrate the presence of glycoproteins within our DIG-reactive protein fractions. These data also demonstrate the presence of a variety of sugars on *H. pylori* glycoproteins, which may arise from the PA pathway (PA derivatives), the LPS biosynthesis pathway (Kdo) or also from a novel pathway (Bac derivatives).

## Discussion

### Protein glycosylation in *H. pylori* extends well beyond the flagellins

The pathogenesis of *H. pylori* is not well understood despite abundant research on its virulence factors. The data presented herein show that the PA biosynthesis pathway, which was hitherto thought to be exclusively devoted to flagellin glycosylation, affects the production of other virulence factors, including lipopolysaccharide and urease. While interconnection between protein glycosylation and LPS synthesis pathways has been reported before in several cases [72], [73], [74], to the best of our knowledge, the genes responsible for the synthesis of sugars used for protein glycosylation and LPS synthesis were shared in all examples. In the current study, the sugar synthesis genes are distinct in each pathway (Fig 1) and therefore, establishing a link between both pathways is not quite as straightforward. This led us to propose that proteins involved in virulence factor production – including LPS - may be glycosylated in a PA-dependent manner (Fig 1), and that glycosylation may be important for their activity.

Our data clearly support the existence of multiple glycoproteins beyond the flagellins, and therefore suggest that protein glycosylation is not limited to flagellins in *H. pylori*. We were able to demonstrate glycosylation of several novel proteins, and several other GPs of lower abundance were also detected by DIG labeling and ProQ-emerald staining and remain to be identified. Aside from this study, the only hint that *H. pylori* may produce multiple GPs was recently published based on a global metabolic profiling study [75], but the GP candidates were neither isolated or characterized, and the actual presence of sugars was not demonstrated. It was also proposed earlier that RecA may be post-translationally modified, potentially through glycosylation, although no direct evidence was provided (no glyco-specific stain and no sugar information) [76]. Therefore, our study is the first to demonstrate labeling of multiple GP candidates by glyco-specific stains and to provide unambiguous MS-based proof of the existence of the GP-associated sugars for non-flagellar proteins in *H. pylori*.

Despite its very small genome, *H. pylori* appears to devote considerable resources to protein glycosylation. This may pertain to the particularly inhospitable environment in which *H. pylori* resides, or to its ability to cause chronic infections. Beyond the direct role of glycosylation on virulence factor production, glycosylation may allow masking of antigenic epitopes of OM proteins, thereby minimizing immune responses and contributing to the chronicity of *H. pylori* infections.

### Identity of the novel *H. pylori* GP candidates

MS analysis of ProQ-emerald-sensitive GP candidates from 2D SDS-PAGE gels led to the identification of 9 candidate GPs. Note that identification was only done for the most abundant GP candidates and that ProQ-emerald staining may not detect all GPs, depending on the nature of their sugars. The identified proteins are either cytoplasmic or associated with the inner membrane, suggesting that protein glycosylation may be important for bacterial physiology and not only for direct interactions with the host. Glycosylation of proteins associated with inner membrane has also been reported recently for *Bacteroides fragilis* [21], [77]. Two of the identified *H. pylori* GP candidates were for two soluble subunits of the general F0F1 ATPase (α and β subunits), which was also amongst the hits obtained from an excised band for a DIG-labeled soluble GP candidate (Band S2, fraction C, Fig 6). Although there is no known precedent for this, this suggests that the *H. pylori* F0F1 ATPase is glycosylated, and that glycosylation may be important for its function. This may reflect a specific ability of *H. pylori* to survive in an acidic environment. Tagging of the identified GP candidates is underway to allow their purification and direct analysis of glycopeptides by MS.

One abundant glycoprotein candidate that reacts strongly with Biotin-hydrazide in a periodate oxidation–dependent manner and appears to have a heterogeneous glycosylation was identified unambiguously as catalase. This finding of catalase glycosylation has never been reported before and studies geared at mapping the glycosylation sites via glycopeptide analyses will be undertaken to allow determining the role of glycosylation on catalase production, stability or function. The low abundance of the other soluble or membrane GP candidates, or their contamination by non-glycosylated proteins has prevented us from identifying them unambiguously. Their identification will require direct analysis of their glycopeptides, which will also map their glycosylation site. Based on the observation that the PA mutants show altered or no O-antigen, it is anticipated that LPS biosynthetic enzymes may be glycosylated by the PA pathway. Therefore, these low abundance GP candidates may comprise LPS biosynthetic enzymes.

Note that periodate-based staining of glycoproteins is prone to non-specific staining. The issue was taken into account in our analyses of the ProQ-emerald-stained proteins and in the sample preparation and analytical methods. All labeling and blotting reactions comprised appropriate negative controls, and the signals for the soluble fraction were obtained on thoroughly delipidated fractions to exclude the presence of contaminating LPS. The samples had undergone repeated rounds of ulcentracentrifugation, as well as precipitation of proteins by ammonium sulfate. Moreover, it was verified by proteinase K treatment and silver staining that the observed signals arose from proteins.

### Identity of the novel *H. pylori* sugars present on GP candidates

The MS identification of the sugars extracted from the GP candidates was a further demonstration that the signals obtained by labeling of the GP candidates with glycan-specific stains were genuinely indicative of protein glycosylation. In terms of the sugars identified, the presence of PA derivatives is consistent with the PA-dependency of one of our GP candidates (band S1). Of note is that only Pse5Ac7Ac has been described for *H. pylori* to date [78]. The presence of the Pse5Am7Ac derivative as identified in this study demonstrates that *H. pylori* has the ability to further modify its PA. Note that the MS/MS spectra are consistent with these assignments for PA derivatives but determining the absolute configuration of these sugars would require investigations by NMR. Moreover, the discovery of Bac derivatives in *H. pylori* is entirely novel as Bac has never been reported in *H. pylori*. Bac is used for protein glycolysation in *C. jejuni* [26] and, like for PA, its biosynthesis is initiated by C6-dehydration of UDP-GlcNAc [32], [79]. Genome mining did not reveal any other UDP-GlcNAc dehydratase beyond FlaA1 in *H. pylori*. This suggests that FlaA1 is shared by both pathways (PA and Bac synthesis) in *H. pylori.* This is consistent with our biochemical data [31]. Note that the Bac-containing fraction (Fraction D) contained several candidate GPs, including the most reactive S4 and S7 and the faint S2 and S5. S4 and S2 were still DIG-reactive in the absence of FlaA1. This would argue that their glycosylation is not FlaA1 dependent. S5 and S7 were only present at very low levels in WT samples, and significant detection of S7 was only observed after enrichment by ion exchange chromatography. In contrast to the WT, bands S5 and S7 were abundant in the *flaA1/HP0366* mutation background, suggesting the existence of an alternate glycosylation pathway that gets up-regulated when FlaA1 or HP0366 is inactivated. The presence of this alternative pathway could lead to heterogeneous glycosylation in the WT strain. Based on our MS-based observation that 1D bands comprise several proteins each, it is possible that the DIG signals from a 1D band arose from protein(s) carrying sugars dependent on FlaA1 (such as Bac) and protein(s) carrying sugars independent from FlaA1. The presence of the latter sugars would lead to an apparent non-dependence on FlaA1 of the glycosylation of all the proteins comprised within the 1D band.

In *C. jejuni*, a Bac derivative (Bac2Ac4Ac) is present on GPs at the base of a heptasaccharide that also comprises GalNAc and Glc [26]. In our samples, the Bac derivatives were also found in association with hits for hexoses, hexosamines and N-acetyl hexosamines, which suggests that *H. pylori* may also be able to assemble a *C. jejuni*-like complex polysaccharide for protein glycosylation.

Apart from Kdo, none of the identified sugars are part of the LPS, which further reinforces the notion that the sugars stem from glycoproteins. However, as explained above, the observation that the PA pathway mutants produced altered LPS led us to examine whether protein glycosylation may affect LPS synthesis. A further potential connection between the LPS machinery and protein glycosylation comes from our observation that Kdo was found associated with our GP candidates. All precautions were taken to prevent contamination of the samples by LPS, and the fractions do not contain any of the other LPS signature sugars, therefore reinforcing the notion that the observed Kdo was bound to a GP. This could indicate that *H. pylori* can also use the LPS synthesis machinery to glycosylate proteins, as seen in other bacterial species. *H. pylori* is known to remove a Kdo residue from its LPS via a dedicated Kdo hydrolase [80], [81], but the fate of the removed Kdo is unknown. We will therefore examine whether the extracted Kdo can be transferred onto the GP candidates, and what is the role of this process.

### Presence of multiple glycosylation pathways in *H. pylori*

The sugar identifications and comparisons of GP candidate profiles between WT *H. pylori* and PA mutants suggest that *H. pylori* harbours several glycosylation pathways: the classical PA pathway that generates the PA derivatives highlighted by MS analyses, a pathway that branches downstream of FlaA1 but upstream of HP0366 and likely generates the Bac derivatives as per analogy with the *C. jejuni* Bac synthesis pathway, and a totally PA-independent pathway that generates the other sugars identified. The mapping of the glycosylation sites of our GP candidates will determine whether the new *H. pylori* glycosylation pathways highlighted by our data involve N- or O-glycosylation. While eukaryotic N-glycosylation requires the NxS/T sequon, an extended consensus sequon D/ExNxS/T was identified for bacterial N-glycosylation [82], [83] but is not absolute [84], [85]. Several of the GP candidates identified so far by MS from ProQ-emerald stained gels harbor a general (eukaryotic like) N-glycosylation sequon (Table 2), but none harbored an extended bacterial one.

In conclusion, this is the first report of the existence and identification of multiple GP candidates and multiple glycosylation pathways in *H. pylori*. These pathways result in the production of several novel sugars never previously demonstrated in *H. pylori* and that could be putatively identified via MS/MS after extraction from DIG-reactive delipidated protein fractions. While no direct assignment of the novel sugars to a specific glycoprotein candidate could be established at this stage, the *a priori* knowledge gained on the glycosylation pathways present in *H. pylori* based on the identification of the extracted sugars should help characterize the new glycosylation pathways biochemically and their targets in the future. We have identified an alternative aminotransferase that may participate in one of the new glycosylation pathways and will investigate its effects both via a biochemical and mutagenesis approach. This will also allow assessing the relative importance of the various glycosylation pathways in *H. pylori*. Likewise, the mapping of the glycosylation sites of our GP candidates will be performed to allow the investigation of the role of protein glycosylation on the function of select proteins via mutagenesis of their glycosylation sites, and on the pathogenicity of the bacterium via analysis of virulence factor production in these glycosylation deficient mutants.

## Materials and Methods

### Bacterial growth conditions

*H. pylori* strain NCTC 11637 (aka ATCC 43504) was a kind gift from Dr S. Logan (NRC, Ottawa, Canada) and corresponds to the original isolate described in [86], [87]. The bacteria were routinely grown to confluence on agar plates containing 37 g/l Brain Heart Infusion (BHI) media (Becton Dickinson), 2.5 g/l yeast extract (YE) (Bioshop), 0.05% sodium pyruvate, 10% horse serum, and the antibiotics amphotericin B (4 µg/ml), trimethoprim (5 µg/ml) and vancomycin (10 µg/ml) under microaerophilic conditions (5% O_2_, 10% CO_2_, and 85% N_2_) at 37^o^C. When necessary, selection with 5 µg/ml kanamycin was applied.

*Escherichia coli* (DH5α) was grown at 37°C in Luria-Bertani medium with selection with 100 µg/ml ampicillin or 30 µg/ml kanamycin when necessary.

### Preparation of the *hp0366* knockout mutant

To prepare the knockout construct, the *hp0366* gene was first amplified by PCR from genomic DNA of *H. pylori* strain 26695 using Expand polymerase (Roche Diagnostics) under conditions recommended by the manufacturer. The primers used were HP0366P5 AGGGTCCATGGGTTTGAAAGAGTTTGCTTATAGC and HP0366P2 GCGTCGGATCCTCATTCTATTTTAAAACTC, which were designed based on genomic data [88] and contained *Nco*I and *BamH*I sites (underlined), respectively. The PCR product was cut with *Nco*I and *BamH*I and inserted into the pET23 derivative [89] that had been previously cleaved with the same enzymes. After transformation in *E. coli* DH5α and selection on ampicillin 100 µg/ml, the plasmid DNA was recovered and the gene was fully sequenced. Sequencing was performed at the Robarts DNA sequencing facility (London, Ontario).

Inverse PCR was then performed on the pET construct described above using primers HP0366P3 CGCTCTCATGGCATGCTC and HP0366P4 CTGTTAAACACTAACGCATG and *Pfu* polymerase (Stratagene) following the manufacturer's recommendations. The PCR product was ligated with a kanamycin resistance cassette that had been PCR amplified from the pHel3 vector (obtained from Dr R. Haas) [90] as described before [10]. The construct was introduced into *H. pylori* strain NCTC 11637 by electroporation [8], [52] and mutant candidates were selected in the presence of 5 µg/ml kanamycin. The mutants were checked for proper gene disruption by PCR and Southern blotting using standard procedures.

### Preparation of the complemented strains

The *hp0366* gene and its promoter was PCR amplified from chromosomal DNA from strain 26695 using primers HP0366P2 (see above) and HP0366P1 GAAGTGGAGGATAAGATG and cloned into the *BamH*I and *EcoR*V sites of the pHel2 shuttle vector (obtained from Dr R. Haas) [90] using standard procedures. The construct was introduced into the wild-type strain NCTC 11637 or *hp0366* mutant strain by electroporation as described previously [10]. The plasmid was extracted from *H. pylori*, transformed into calcium chloride competent *E. coli* DH5α, extracted again and checked for integrity by restriction digestion to allow for the selection of complemented strains worthy of further phenotypic characterization.

### Lipopolysaccharide (LPS) analyses

LPS was prepared according to [71]. Briefly summarized, cell pellets (50 µl, wet cell volume) were resuspended in 200 µl of 1 M Tris buffer, pH 6.8 containing 2% SDS, 4% β-mercaptoethanol and 10% glycerol. After boiling for 30 min, the samples were treated for 1 h with 100 µg of proteinase K at 56^o^C. The samples were then analyzed on 15.6% SDS-PAGE gels and detection was performed by silver staining [91].

### Electron microscopy (EM)

The *H. pylori* cells were harvested from BHI plates and stained with 2% ammonium molybdate for EM analysis. EM was done at the EM facility of the Department of Microbiology and Immunology at the University of Western Ontario.

### Urease activity measurements

*H. pylori* pellets of the wild-type NCTC 11637 strain, the *hp0366* mutant and the complemented strain were dissolved in breaking buffer (20 mM sodium phosphate, pH 7.4, 1 mM EDTA) and lysed by mechanical disruption using glass beads. After centrifugation for 5 min at 12,000 x g, the supernatant was removed and its soluble protein content was measured using the Biorad protein determination reagent. The supernatants were diluted to 0.9, 1.8 and 2.7 g/l in breaking buffer and assayed for urease production using phenol red as an indicator [92]. Briefly summarized, 10 µl of supernatant at 0.9, 1.8 or 2.7 g/l in total proteins were added to 100 µl of 0.33 mM urea, 0.001% phenol red in 5 mM sodium phosphate at pH 6.7, supplemented with 0.15 M NaCl. The OD_565nm_ which indicates urease activity, was measured at regular intervals over the course of two hours. Because of the normalization of the samples according to protein content, direct comparison of the OD_565nm_ values provides a relative comparison of the levels of urease activity produced by the WT and the mutant, although the absolute levels of urease activity was not calculated. Measurements were done in triplicates for each supernatant protein concentration.

### Tissue culture experiments

Gastric epithelial cells (AGS cells) were kindly provided by Dr P. Sherman (Hospital for Sick Children, Toronto). Approximately 300,000 cells per well were grown for ∼24 h in 24-well plates until confluent, reaching about 600,000 cells per well. The cells were infected for 5 h with wild-type NCTC 11637 or mutant *H. pylori* that had been grown for 48 h on BHI plates. Approximately 6×10^7^ cfus of *H. pylori* were added, resulting in a multiplicity of infection of 1 AGS cell per 100 bacteria. The plates were spun briefly (5,000×g for 5 min at room temperature) to maximize contact between the bacteria and the cell monolayer. To determine total bacterial cell association (adhering and internalized bacteria), the AGS cells were washed 3 times, lysed with 0.08% saponin (Sigma) for 10 min and viable bacterial counts were determined by plating serial dilutions. To determine the number of internalized bacteria, the AGS cells were treated with 200 µg/ml gentamycin for 2 h to kill extracellular bacteria. The cells were then washed and treated as above to determine bacterial viable counts. Three independent sets of experiments were done, with triplicates within each experiment. The data represent the average of all experiments. The data were analyzed by unpaired t-test with P<0.001 as the limit for significance.

### 2D gel electrophoresis and ProQ-emerald staining

*H. pylori* cells grown on BHI plates for 2 days were harvested by centrifugation, washed in saline and kept frozen at −20°C until needed. A cell pellet containing ∼0.5 mg of proteins (as per Biorad protein determination assay) was resuspended in 100 µl lysis buffer (30 mM Tris-HCl, pH 8.8, 2 M thiourea, 7M urea and 4% CHAPS) and sent to Applied Biomics (California, project manager Dr G. Fu) for labeling and 2D gel analyses. Total proteins (0.5 mg) of *H. pylori* were labeled with the Cy5 dye (280 pmol) for 30 min to achieve minimal labeling of proteins (1 labeled lysine per protein on average). The reaction was quenched by addition of 7 nmol of lysine. The 2D gels (pH 3 to 10, 12% gel) were loaded with a mixture of 30 µg of Cy5-labeled proteins with 120 µg of unlabeled sample that had been dissolved in lysis buffer. Total proteins were detected in the Cy5 channel (excitation 650 nm, emission 670 nm). Staining with ProQ Emerald 488 (Invitrogen) was performed according to supplier's instructions, and the ProQ-emerald stain was detected at 520 nm (excitation at 510 nm). The DeCyder software was used to detect and quantitate the Cy5-labeled and ProQ-reactive spots. Background intensities for each signal were subtracted from the signal of each spot before the ProQ/Cy5 signal ratios were calculated. An unpaired t-Test was performed for statistical analysis of the GP candidates (ProQ/Cy5 ratio >0.17) versus non GP proteins (equal variance for both populations, two-tailed p value, significance set at p<0.001).

### In-gel Digestion, Mass Spectrometry, and Database search for ProQ-emerald-reactive GP candidates

These experiments were also performed by Applied Biomics (California). Proteins of interest were digested in-gel with modified porcine trypsin protease (Trypsin Gold, Promega). The digested tryptic peptides were desalted by Zip-tip C_18_ (Millipore). Peptides were eluted from the Zip-tip with 0.5 ul of matrix solution (alpha-cyano-4-hydroxycinnamic acid (5 mg/ml in 50% acetonitrile, 0.1% trifluoroacetic acid, 25 mM ammonium bicarbonate)) and spotted on the MALDI plate (model ABI 01-192-6-AB). MALDI-TOF mass spectrometry (MS) and TOF/TOF tandem MS/MS were performed on an ABI 4700 mass spectrometer (Applied Biosystems, Framingham, MA). MALDI-TOF mass spectra were acquired in reflectron positive ion mode, averaging 4000 laser shots per spectrum. TOF/TOF tandem MS fragmentation spectra were acquired for each protein, averaging 4000 laser shots per fragmentation spectrum on each of the 10 most abundant ions present in each sample (excluding trypsin autolytic peptides and other known background ions).

Both the resulting peptide mass and the associated fragmentation spectra were submitted to GPS Explorer workstation equipped with MASCOT search engine (Matrix science) to search the National Center for Biotechnology Information non-redundant (NCBInr) database. Searches were performed without constraining protein molecular weight or isoelectric point, with variable carbamidomethylation of cysteine and oxidation of methionine residues, and with one missed cleavage allowed in the search parameters. Candidates with either protein score C.I.% or Ion C.I.% greater than 95 were considered significant.

### MS identification of proteins from the soluble protein fraction (SPF)

GP candidate bands were cut out from Coomassie stained SDS-PAGE gels and subjected to in-gel trypsin digestion. The extracted peptides were analyzed by LC-MS/MS using standard procedures at the Don Rix Mass spectrometry facility of the University of Western Ontario.

### RNA isolation

Total RNA isolation was performed with the GE Healthcare RNAspin Mini RNA isolation kit. The cells were harvested directly in the lysis buffer provided by the manufacturer and isolation was carried out as recommended by the manufacturer except that lysozyme-mediated lysis was performed. The lysate was filtered and loaded onto the RNA-binding column. Contaminating DNA was removed by on-column treatment with DNAseI and total RNA was eluted in RNAse free water. An additional round of DNAseI treatment was performed off column using RNase-free DNaseI (from Roche) according to supplier's instructions. The treated RNA was quantitated in a SmartSpec 3000 spectrophotometer by reading the absorbance at 260 nm, and using A_260_ = 1 for 40 µg/ml as a reference. The RNA was stored at −20°C until needed.

### Reverse-transcription

The isolated total RNA was reverse transcribed into cDNA using iScript^TM^cDNA Synthesis Kit (Biorad). Reverse transcription was done with random hexamer primers in order to allow downstream amplification of all genes of interest from a single cDNA preparation. Following the manufacturers instructions and using a total volume of 40 µL, total cDNA was synthesized from 30 µL of RNA, diluted 1/10 or 1/100, using iScriptRNaseH^+^ reverse transcriptase. Negative controls were also created using RNase-free water instead of the reverse transcriptase.

### qRT-PCR

The sequence of the primers used for qRT-PCR are indicated in Table 1. Each reaction contained 2.25 µL cDNA, 7.5 µL 2X Perfecta SYBR Green SuperMix (Quanta Biosciences), and 0.67 µL each of 6.67 pmol/μL forward and reverse primers in a total reaction volume of 15 µL. Real time analysis was done with a Rotor-Gene 6000 Series 1.7 (Corbett). Real-time PCR conditions were: initial denaturation and enzyme activation at 95°C for 10 min, followed by 45 cycles of denaturation at 95°C for 15 s, primer annealing at 52°C for 45 s, and elongation at 60°C for 60 s. The last cycle was followed by a final denaturation at 95°C for 15 s, and acquisition of a melt curve starting with 60°C for 60 s, and raising the temperature every 15 s by 0.3°C to a final temperature of 95^o^C. A fluorescence reading was taken after each increase.

**Table 1:** List of genes whose expression was investigated by qRT-PCR.

Gene	Function	Primer names	Primer sequences
UreA	Urease A (HP0073, of *ureA-B*	HP0073P1	GTTAGACAAGTTGATGCTCC
	operon)	HP0073P2	CCATCAGGAAACATCGCTTC
UreI	Accessory protein for urease	HP0071P1	CCACCCTACAGCCCCTG
	(HP0071of *ureIEFGH* operon)	HP0071P2	CGCAGCAGGAATCGTGTTG
FucST	Fucose synthase (HP0044)	HP0044P5	(AAGACGGATCC)ATGAAAGAAAA
			AATCGCTTTAATCAC
		HP0044P7	CTGTAGGCTTAGTGGTAGCGA
FucT	α-1,3- fucosyl transferase	HPFucTP1	CTGGGGGAGGAGCCGTG
	(HP0379)	HPFucTP2	GGGTGCGTGTGCAAGTATC
GalT	β-1,4- galactosyl transferase	HP0826P1	GCCACCCACCACCAAGCGC
	(HP0826)	HP0826P2	CACCCAAGCTCCCCCAAG
WaaL	O-antigen ligase (HP1039)	HP1039P9	(GGAATTC)GTGTTGAAAGAGCGTT
			TGAAAGCC
		HP1039P4	(GAAGATCT)AAACATGTTAGGGAA
			GATGCT
FlaA	Flagellin A (HP0601)	FlaA5	ATGGCTTTTCAGGTCAATAC
		FlaA4	CATCCATAGCCTTATCCGC
FlaB	Flagellin B (HP0115)	HP0115P1	GGATAAATACCAATATCGCC
		HP0115P2	CATCCATCGCTTTATCTGC
FlhA	Basal body and transcriptional	HP1041P1	GGGCCTAGTGCGGTGAG
	regulator (HP1041)	HP1041P2	CCGCATCAATCGCCATTTG
CoAST	AcCoA synthetase (HP1045)	HP1045P1	GTCATTATCTATATGCCCAT
		HP1045P2	CTGGCTTGAGCATGTAAGG

The functions of the genes are indicated, as well as the names and sequences of the primers used. Nucleotides in brackets correspond to polynucleotide tails and restriction sites included for other cloning purposes.

### Culture conditions and preparation of soluble and membrane protein fractions

Bacteria grown on BHI plates for 48h were harvested in 1 ml of cold PBS (10 mM sodium phosphate, 140 mM NaCl) per plate. The bacteria were spun down at 4,000*xg*. for 15 min at 4^o^C, and gently resuspended in the same volume of cold PBS. The optical density at 600 nm of the suspension was taken on a SmartSpec 3000 spectrophotometer (Bio-Rad) and normalized to 0.6. The suspended bacteria were aliquoted into 12 ml fractions, representing 10–13 plates of growth, re-pelleted, and the cell pellet was frozen at −20°C for future protein analysis.

Each pellet was resuspended in 12 ml of Tris buffered saline (TBS: 100 mM Tris pH 8.5, 50 mM NaCl). The cells were lysed in a French press cell (GlenMills) at 1500 psi on ice. Cellular debris were pelleted (14,000*xg*, 30min, 4^o^C). The supernatant was centrifuged at 100,000*xg* for 80 min at 4°C in an Optimax ultracentrifuge with a TLA-110 rotor (Beckman Coulter). The supernatant containing the soluble proteins was transferred to new tubes, and the ultracentrifugation was repeated. The final supernatant was reserved as the soluble protein fraction (SPF). The pellet from the first ultracentrifugation step, containing membrane proteins, was resuspended in TBS buffer and the ultracentrifugation was repeated. The pellet that had been ultracentrifuged twice was washed in TBS buffer and reserved as the membrane protein fraction (MPF).

### Inner and outer membrane preparation

Wild-type *H. pylori* was grown for 2 days on 45 BHI plates, as described above. The cells were harvested in saline (0.85% NaCl), spun down at 4,000*xg* for 30 min at 4°C and re-washed once in 25 ml of saline. The pellet was resuspended in saline to have an OD_600_ = 5. The cells were lysed by passage through a French press (GlenMills) four times at 1500psi on ice. Cellular debris were pelleted by centrifugation at 5,000*xg* for 1 h at 4°C. Insoluble proteins were removed from the supernatant by a 1 h spin at 13,000*xg* at 4°C. Membrane proteins were pelleted from the supernatant by ultracentrifugation at 100,000*xg* for 1 h at 4°C. The membrane pellet was washed in 500 µl saline by ultracentrifugation in a micro-ultracentrifuge (Optimax ultracentrifuge, Beckman Coulter, TLA-110 rotor). To solubilize inner membrane proteins, the membrane pellet was resuspended in 300 µl of PBS (45 mM sodium phosphate, 135 mM NaCl, pH 7.2) with 1% N-lauroylsarcosine (Sigma) and mixed on a nutator for 1 h at room temperature. Outer membrane proteins were pelleted down by ultracentrifugation at 100,000*xg* for 1 h at 4°C. To refine the separation, the supernatant containing inner membrane proteins was ultracentrifuged 3 more times until no pellet was visible. Similarly, the outer membrane pellet was subjected to another around of N-lauroyl sarcosine treatment and ultracentrifugation to remove contaminating IM proteins. The final outer membrane pellet was resuspended in 300 µl MilliQ water.

### DIG-hydrazide labeling of immobilized glycoproteins

The protein content of samples was assessed by Bradford assay (Biorad reagent). Samples were normalized to 20 µg of proteins, separated by 1D SDS-PAGE and transferred to nitrocellulose membrane. The membrane was washed 3 times in 50 ml of PBS (50 mM sodium phosphate, 150 mM NaCl, pH 6.5) for 10 min each. The glycans were oxidized by covering the membrane in 20 ml of 10 mM sodium periodate in 100 mM sodium acetate pH 5.5 in the dark for 20 min. The residual sodium periodate was washed off with a second series of 3 washes with 50 ml of PBS. To label the proteins, 10 ml of 1 µM DIG-hydrazide (digoxigenin-3-O-succinly-ε-aminocaproic acid hydrazide, Roche) in 100 mM sodium periodate pH 5.5 was added and incubated for 1 h. Residual DIG-hydrazide was neutralized and removed by 3 washes with 50 ml of TBS (50 mM Tris, 140 mNaCl, pH 7.2). All procedures were carried out at room temperature.

### DIG-hydrazide labeling of glycoproteins in solution

Approximately 20 µL of 1 µg/µL protein solution was used per in-solution labeling reaction. The labeling was perfomed in 0.1M sodium acetate pH 5.5, with oxidation by incubation with 1.6 mM sodium periodate for 20 min at room temperature in the dark. The reaction was stopped by addition of 20 mM (final concentration) of sodium disulfite, and incubating for 5 min at room temperature. DIG-labeling of the proteins was performed by addition of 1 mM (final) DIG-hydrazide. After 1 h incubation at room temperature, the reaction was stopped by neutralizing the pH with the addition of ¼ V of 4X SDS-PAGE loading buffer. The proteins were separated by 1D SDS-PAGE gel, transferred on nitrocellulose for anti-DIG Western blot.

### Detection of DIG-labeled glycoproteins by anti-DIG Western blot

The membrane was washed in de-ionized water and total proteins were stained by PonceauS. The membrane was de-stained in TBS, and blocked with 20 ml of 10% (w/v) skim milk for 1 hour at room temperature and then refrigerated overnight. The membrane was washed twice for 10 min each in 50 ml of TBSTT (50 mM Tris, 140 mM NaCl, 2% Triton X-100, 0.5% Tween-20, pH 7.2), once for 10 min in 50 ml of TBS and then equilibrated for 10 min in 50 ml of PBS. The membrane was probed with 20 ml of 1∶5000 mouse anti-DIG primary antibody (Roche) and by anti-mouse AlexaFluor-680 conjugate secondary antibody (Molecular Probes) in PBS for 60 min in the dark. The membrane was washed twice in 50ml of TBSTT for 10 min and once in 50 ml of TBS for 10 min. The membrane was scanned at 700 nm on a Licor Odyssey infra-red scanner.

### Biotin-hydrazide labeling of glycoproteins

As commercial supplies of DIG-hydrazide were discontinued during the course of these studies, labeling of GPs was also performed using biotin hydrazide (BHz). Samples from cell fractionation were diluted 1∶1 in 200 mM sodium acetate, pH 5.5. Samples were oxidized using 10 mM sodium periodate (Sigma) for 20 min at room temperature in the dark. The reaction was stopped by addition of 16 mM meta-bisulphite solution made in 200 mM sodium acetate, pH 5.5 and incubated at room temperature for 5 min. The labeling reaction was performed by adding biotin hydrazide (Sigma) to 0.1 mM and the mixture was incubated at room temperature for 1 h. The labeled samples were separated by SDS-PAGE and transferred onto nitrocellulose. The membrane was blocked with 10% Horse Serum (Gibco) in MilliQ water, and the biotin-labeled GPs were detected by probing the membrane with 10 ml of 1 µg/ml streptavidin-AlexaFluor-680 conjugate (Molecular Probes) in PBS for 30 minutes in the dark. The membrane was washed twice in 50 ml of TBSTT for 10 min and once in 50 ml of TBS for 10 min. The membrane was scanned at 700 nm on a Licor Odyssey infra-red scanner to detect the fluorescent streptavidin conjugate.

### Phenyl boronate enrichment of glycoproteins

The membrane protein fraction (MPF) obtained as described above from a 12 ml *H. pylori* suspension at OD_600_ of 0.6 was solubilized in 12 ml of 2% Triton-X 100 in PBS. The MPF was filtered through a 0.45 µm filter and was loaded on a 2 ml Prosep PB (phenyl boronate, Millipore) affinity column. Before use, the PBA (phenyl boronate affinity) matrix was flushed with water, 5 column volumes (CV) each of 0.1 M acetic acid, water, 8 M Urea, water, 0.1 M acetic acid in 20% ethanol and finally washed in 5 CV of running buffer (0.5% Triton-X 100 in PBS). The column was washed with 5 CV of running buffer, and elution was performed with 3×1 CV of 0.2 M Tris buffer (pH 7.4) containing 0.5% triton for 5 min each. The fractions were concentrated by ammonium sulfate precipitation (see below) before SDS-PAGE analysis and Western blotting.

### Ion exchange chromatography

The soluble protein fraction (SPF) of WT *H. pylori* was fractionated by strong anion and cation exchange chromatography on a Mono Q 5/50 GL and Mono S HR 5/50 columns (1 ml CV, 5 mm×50 mm; GE Healthcare). Columns were installed on an AKTA Explorer FPLC equipped with a 10 ml Super Loop (GE Healthcare). The system was operated at a flow rate of 1 ml / min. First, the SPF was run through the MonoQ column. The MonoQ column was equilibrated for 10 CV with loading buffer (50 mM NaCl in 20 mM Tris pH 8.5) prior to sample injection. The samples were eluted with a 10 CV isocratic gradient with loading buffer followed by a 40 CV linear gradient to 100% elution buffer (1M NaCl in 20 mM Tris pH 8.5). The flow through collected during anion exchange chromatography was dialyzed in 50 mM NaCl in 20mM sodium phosphate pH 6.5, and fractionated on the MonoS column that had been equilibrated in the same buffer. The samples were eluted with 10 CV of loading buffer followed by a 40 CV linear gradient to 1 M NaCl in 20 mM sodium phosphate pH 6.5.

### Ammonium sulfate precipitation of protein containing samples

Each 1 ml protein fraction obtained from ion exchange chromatography was brought to saturation with the addition of 0.8 g ammonium sulfate. After full dissolution of the ammonium sulfate crystals, protein precipitation was allowed to occur for 2 h at 4^o^C. The samples were pelleted at 14,000*xg* for 30 min, and the precipitate was resuspended in 1ml of water.

### Release of monosaccharides from glycoproteins by acid hydrolysis

These samples were normalized to 1 g/l total protein as assessed by Bradford assay (Bio-Rad). The monosaccharides from glycan-containing proteins were cleaved off with 2 M trifluoroacetic acid (TFA) at 110°C for 4 hours as described previously [65], [66]. The TFA was removed in a speed-vac (Eppendorf), the volume was adjusted to 1 ml with water, and the hydrolyzed sugars were separated from the proteins by ultrafiltration through a Nanosep Omega centrifugal device (Pall) with a 3.5 kDa cutoff. The sugars were then analyzed by High Performance Anion Exchange with pulse amperometric detection (HPAE-PAD) and ESI-MS/MS.

### HPAE-PAD analysis

These analyses were carried out on an ICS-3000 ion chromatography system (Dionex) equipped with an electrochemical detection cell with AgCl reference electrode operated with a quadraform potential waveform specific for sugar detection. The column was a 4x50 mm CarboPac PA1 column (Dionex) operated at 30°C and 1 ml / min. The system was operated with Chromeleon software. The column was washed for 20 min with 200 mM NaOH, and equilibrated for 20 min with 16 mM NaOH. Ten µl of sugar samples (adjusted to 16 mM NaOH in water) were injected, and elution was performed by a 20 min isocratic gradient of 16 mM NaOH, followed by a 20 min linear gradient of 0 mM to 320 mM sodium acetate in 16 mM NaOH and a 10 min linear gradient from 320 mM to 1 M sodium acetate and from 16 mM to 100 mM NaOH. Sugar standards were 100 µM solutions of α-L (+) fucose (Sigma), D (+) mannose (Sigma), D (+) glucosamine hydrochloride (Nutritional Biochemicals Corporation), and D (+) dextrose (Sigma).

### Analysis of released glycans by LC-ESI MS/MS

Samples were analyzed by MS at the Don Rix proteomics facility of the University of Western Ontario. The LC-MS/MS system consisted of a Waters CapLC with a reversed phase Phenomenex Jupiter Proteo 90 A column (150×1.0 mm, 4 µm) coupled to a Q-TOF (micro, Waters) mass spectrometer. Online LC was performed with 0.01% TFA in water (Solvent A) and 95% acetonitrile with 0.01% TFA (Solvent B). Following injection of 5 µL of sample, the sugars were separated with a 30 min linear gradient from 1% to 50% B followed by a 15 min gradient from 50% to 100% B. Finally, an isocratic gradient of 100% B was held for 10 min. LC-ESI-MS analysis was performed in both the positive ion mode and the negative ion mode with an orifice voltage of 80 V and a scan range of 200–800 m/z. Collision-induced dissociation was performed with argon as the collision gas and a variable collision energy of 20–30 eV over a mass range of 80–600 m/z. Data were acquired and analyzed by MassLynx 4.0 (Micromass).

### Sugar MS data analyses

The hits obtained in MassLynx 4 were screened for potential sugar hits using GlycoWorkbench, an MS glycan analysis software that allows users to input non usual sugars to the database [69]. Sugars manually added to the database were PA, bacillosamine and legionaminic acid, as well as all their currently known bacterial derivatives. The sugar hits obtained were screened again in MassLynx for LC and MS peak quality to generate a refined list of probable sugars. This list was then used as an inclusion list for LC-ESI MS/MS experiments. For any MS/MS hits, the sizes of the fragments observed in the tandem mass spectrum were calculated and compared to a theoretical fragmentation pattern calculated for the sugar of interest.

## Supporting Information

Figure S1 **Mass spectrometry analysis of the sugars extracted from GP candidates by acid hydrolysis.** The sugars were analyzed by LC-MS/MS. In each panel, the molecular structure of the expected sugar is shown above the MS/MS spectrum, with the expected fragmentation pattern and associated mass loss. The MS/MS spectra are annotated with the total mass loss intervals and with mass loss combinations that lead to the size of the observed peaks. All spectra are shown as sodium adducts. Panel A: Hexose. Panel B: 3-deoxy-D-*manno*-octulosonic acid (Kdo).
(TIF)
