# Supplementary figures and images for: Protein Glycosylation in Helicobacter pylori: Beyond the Flagellins?
Source: PLoS One. 2011 Sep 30;6(9):e25722. doi: 10.1371/journal.pone.0025722 (PMC3184161; doi:10.1371/journal.pone.0025722)

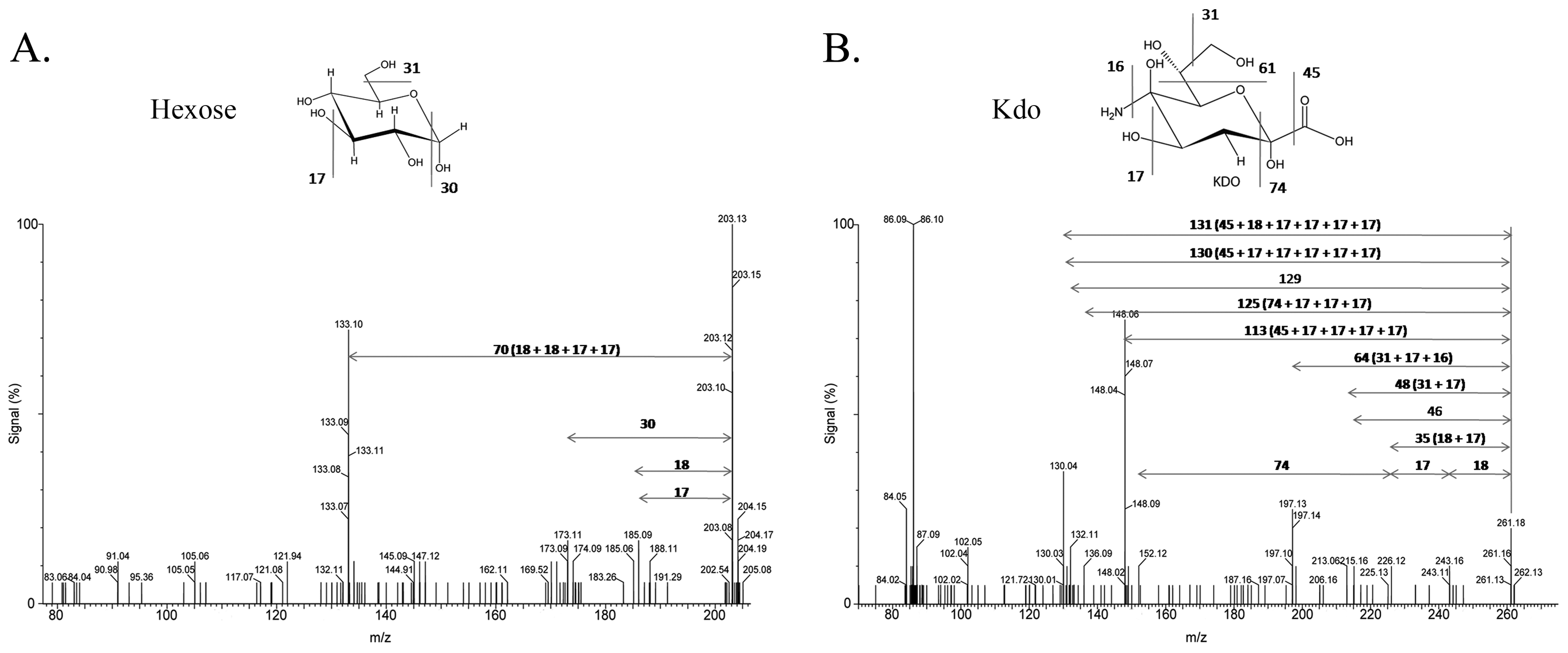

Supplement: Figure S1 — Mass spectrometry analysis of the sugars extracted from GP candidates by acid hydrolysis. The sugars were analyzed by LC-MS/MS. In each panel, the molecular structure of the expected sugar is shown above the MS/MS spectrum, with the expected fragmentation pattern and associated mass loss. The MS/MS spectra are annotated with the total mass loss intervals and with mass loss combinations that lead to the size of the observed peaks. All spectra are shown as sodium adducts. Panel A: Hexose. Panel B: 3-deoxy-D-manno-octulosonic acid (Kdo). (TIF) [file pone.0025722.s001.tif]
